# Transmission of temporally correlated spike trains through synapses with short-term depression

**DOI:** 10.1371/journal.pcbi.1006232

**Published:** 2018-06-22

**Authors:** Alex D. Bird, Magnus J. E. Richardson

**Affiliations:** 1 Warwick Systems Biology Centre, University of Warwick, Coventry, United Kingdom; 2 Ernst Strüngmann Institute for Neuroscience, Max Planck Society, Frankfurt, Germany; 3 Frankfurt Institute for Advanced Studies, Frankfurt, Germany; 4 Warwick Mathematics Institute, University of Warwick, Coventry, United Kingdom; New Jersey Institute of Technology / Rutgers Univ-Newark, UNITED STATES

## Abstract

Short-term synaptic depression, caused by depletion of releasable neurotransmitter, modulates the strength of neuronal connections in a history-dependent manner. Quantifying the statistics of synaptic transmission requires stochastic models that link probabilistic neurotransmitter release with presynaptic spike-train statistics. Common approaches are to model the presynaptic spike train as either regular or a memory-less Poisson process: few analytical results are available that describe depressing synapses when the afferent spike train has more complex, temporally correlated statistics such as bursts. Here we present a series of analytical results—from vesicle release-site occupancy statistics, via neurotransmitter release, to the post-synaptic voltage mean and variance—for depressing synapses driven by correlated presynaptic spike trains. The class of presynaptic drive considered is that fully characterised by the inter-spike-interval distribution and encompasses a broad range of models used for neuronal circuit and network analyses, such as integrate-and-fire models with a complete post-spike reset and receiving sufficiently short-time correlated drive. We further demonstrate that the derived post-synaptic voltage mean and variance allow for a simple and accurate approximation of the firing rate of the post-synaptic neuron, using the exponential integrate-and-fire model as an example. These results extend the level of biological detail included in models of synaptic transmission and will allow for the incorporation of more complex and physiologically relevant firing patterns into future studies of neuronal networks.

## Author summary

Synapses between neurons transmit signals with strengths that vary with the history of their activity, over scales from milliseconds to decades. Short-term changes in synaptic strength modulate and sculpt ongoing neuronal activity, whereas long-term changes underpin memory formation. Here we focus on changes of strength over timescales of less than a second caused by transitory depletion of the neurotransmitters that carry signals across the synapse. Neurotransmitters are stored in small vesicles that release their contents, with a certain probability, when the presynaptic neuron is active. Once a vesicle has been used it is replenished after a variable delay. There is therefore a complex interaction between the pattern of incoming signals to the synapse and the probablistic release and restock of packaged neurotransmitter. Here we extend existing models to examine how correlated synaptic activity is transmitted through synapses and affects the voltage fluctuations and firing rate of the target neuron. Our results provide a framework that will allow for the inclusion of biophysically realistic synaptic behaviour in studies of neuronal circuits.

## Introduction

Variability in synaptic function arises from stochasticity in processes ranging in scale from the transitory opening and closing of ion channels to probabilistic neurotransmitter release and vesicle restock [1–4]. The transmission of signals between neurons is therefore inherently stochastic and, moreover, will interact in a history-dependent manner with the patterns in the incoming presynaptic drive [5–9]. A common approach to treating this stochasticity analytically assumes that neuronal firing is uncorrelated, with a Poisson process typically used to model spike times [10–12]. However, non-Poissonian activity is regularly observed *in vivo* [13–16] and models suggest that, even in the absence of short-term synaptic dynamics, it can have a substantial effect on the propagation of activity [17–21]. It can be expected that the impact of non-Poissonian presynaptic activity will be further complicated when combined with vesicle-depletion depression, in which synaptic transmission becomes weaker and less reliable as stores of available neurotransmitter are depleted and yet to be restocked [22–24].

Transmission through plastic synapses has been shown to decorrelate input spike trains [5, 25, 26], typically increasing the computational power [27, 28] and efficiency [29] of a neuronal network. While average rate effects under the influence of depression have been extensively studied [30–32], a compact set of analytical results for correlated spike trains and stochastic quantal vesicle release from multiple sites has remained elusive (for a full discussion of existing results, see the Discussion). Recently, it has been shown [33] that correlated firing patterns more regular than Poissonian can increase the rate of vesicle release, thereby enhancing the fidelity and efficiency of signal transmission, whilst more irregular spike trains can lead to a decrease in neurotransmitter release [25, 27].

To further analyse how the interaction of correlated presynaptic drive and short-term depression affect quantal synaptic transmission, here we derive a number of analytic results for renewal processes, for which the incoming spike train is fully characterised by the inter-spike-interval (ISI) distribution. This type of presynaptic drive includes that generated by the leaky, quadratic and exponential integrate-and-fire models driven by white-noise [34–36], which are models commonly used to fit experimental data [37–39]. It can be noted that these models will also generate ISIs that are well approximated by renewal processes when the correlations of their incoming synaptic drive are much shorter in time than the typical outgoing ISIs.

We derive equations for the occupancy and the temporal structure of release events when presynaptic cells have spiking patterns fully characterised by their inter-spike-interval distribution. We then show how these can be used to calculate the post-synaptic voltage mean and variance when the presynaptic neurons make multiple independent contacts. These results are illustrated using gamma-distributed ISIs and presynaptic integrate-and-fire neurons. We show that the results allow for a good estimation of post-synaptic firing rates thereby opening the way for the analysis of the role synapses with stochastic short-term depression play in feed-forward and recurrent neuronal networks in which presynaptic spike-trains are typically non-Poissonian.

## Materials and methods

### Synaptic release sites

A quantal model of synaptic dynamics is used where the binary variable *x* represents the occupancy (*x* = 1) of a release site by a neurotransmitter-filled vesicle, with *x* = 0 otherwise. On the arrival of a presynaptic action potential, neurotransmitter is released with probability *p* if a vesicle is present at the site. For brevity, the probability that a present vesicle is not released, 1 − *p*, is written as *q*. Sites that are empty are then restocked at memoryless rate λ (Poisson process). Note that because *x* is a binary variable taking values of 0 and 1, *x*^2^ ≡ *x* and so Var(*x*) = 〈*x*〉(1 − 〈*x*〉) where the notation 〈*X*〉 is used as the expectation of any quantity *X* over all stochastic processes (spike times, release and restock events). Values of parameters used to generate figures, unless otherwise stated, are given in Table 1.

**Table 1:** Parameters and their values used for figures. Parameters are grouped together as: those pertaining to occupancy and release statistics; those required for calculation of the post-synaptic response; and those used to generated ISI statistics. Certain neuronal parameters, such as *τ* and *μ*, are used for both pre and post-synaptic integrate-fire models.

Parameter	Interpretation	Value
*p*	probability of release on arrival of a spike, given vesicle is present	0.6
λ	rate empty release sites are restocked	2Hz
*r*	rate of presynaptic spiking	varies
*τ*	membrane time constant	20ms
*μ*	voltage resting potential in absence of input	varies
*a*	amplitude of EPSP induced by fusion and release of single vesicle	0.3mV
*N*	number of presynaptic neurons	varies
*n*	vesicle release sites per presynaptic neuron	varies
*α*	shape parameter for gamma-generated ISIs	varies
*σ*	voltage standard deviation for presynaptic integrate-and-fire neurons	varies
*v*_re_	voltage reset after an action potential	varies
*v*_th_	voltage threshold at which a spike is registered	10mV (LIF) 15mV (EIF)
*δ*_T_	spike onset range for EIF model	1.5mV
*v*_T_	spike onset threshold for EIF model	10mV

### ISI distribution

The presynaptic spike train is modelled as a renewal process characterised by the ISI distribution *f*(*t*) where the firing rate *r* is the reciprocal of the mean ISI. Though there are no correlations between successive ISIs, the spike train itself will in general be non-Poissonian and can range between bursting and regular extremes: bursting neurons have positively autocorrelated spike trains at short times whereas more periodically firing neurons generate trains that are negatively autocorrelated over short time frames. Many results in this paper will involve expectations over the ISI distribution of exponential functions

$$\begin{array}{c} \left\langle e^{-zt}\right\rangle =\int_{0}^{\infty }f\left(t\right)e^{-zt}dt=L\left(z\right) \end{array}$$ (1)

where *z* can be complex (with restrictions on its real part related to the specific functional form the ISI distribution). This can also be interpreted as the Laplace transform of the ISI distribution, which we will denote as L(z). For Laplace transforms of other quantities, say *X*(*t*), we will use the notation $L_{X}\left(t\right)$. Note that the Fourier transform $\hat{f}\left(\omega \right)$ of the ISI distribution can also be interpreted as an expectation

$$\begin{array}{c} \hat{f}\left(\omega \right)=\int_{-\infty }^{\infty }f\left(t\right)e^{-i\omega t}dt=\left\langle e^{-i\omega t}\right\rangle =L\left(i\omega \right) \end{array}$$ (2)

and is directly related to its Laplace transform, remembering that *f*(*t*) = 0 for *t* < 0. This will allow many existing results from the literature on integrate-and-fire ISI distributions in the frequency domain to be used in the subsequent analyses.

### Gamma-distributed ISI distributions

Gamma distributed ISIs provide a useful illustration of the results presented here as a single shape parameter *α* continuously varies the train between bursting for *α* < 1 and more regular *α* > 1. The ISI distribution *f*(*t*) is given by

$$\begin{array}{c} f\left(t\right)=\frac{{\left(\alpha r\right)}^{\alpha }}{\Gamma (\alpha )}t^{\alpha -1}e^{-\alpha rt} \end{array}$$ (3)

for positive *t* and zero otherwise, where $\Gamma \left(\alpha \right)=\int_{0}^{\infty }t^{\alpha -1}e^{-t}dt$ is the gamma function. When *α* = 1 the ISI distribution becomes exponential and the presynaptic spike train is a Poisson process. The expectation of an exponential function (Eq 1) over this class of ISI distribution is

$$\begin{array}{c} \left\langle e^{-zt}\right\rangle ={\left(\frac{\alpha r}{z+\alpha r}\right)}^{\alpha } \end{array}$$ (4)

where *z* can be a complex constant as long as the real part of *z* + *αr* is greater than zero.

### Data availability

Together with this paper we provide JULIA code in the Jupyter Notebook environment for generating each of the figures shown in the paper. All five scripts are published under the GNU General Public Licence, Version 3 (http://www.gnu.org/copyleft/gpl.html).

## Results

After writing down some general statements for arbitrary spike times, we focus on deriving exact results for the case when correlated presynaptic spiking is a renewal process and fully described by the ISI distribution. We present formulae for the steady-state vesicle-release site occupancy averaged over time as well as its mean value just before the arrival of a presynaptic action potential. We then derive an integral equation for the occupancy at some later time given a release event at an earlier time: this will allow us to calculate the autocovariance of release events which in turn leads to an analytical form for the postsynaptic voltage variance. We then generalise this result to a scenario in which each presynaptic cell makes multiple independent contacts. Because of the shared presynaptic drive across these contacts, an additional level of correlation is generated in the input to the post-synaptic cell. We characterise these correlations through the cross-covariance of release events and extend the formula for the post-synaptic voltage to multiple contacts. These results are illustrated using gamma-distributed ISIs for the presynaptic trains. It is then further shown how all results can be derived exactly for presynaptic LIF models or numerically for other classes of integrate-and-fire model, such as the EIF model that are themselves all driven by Gaussian white-noise drive. Finally, we consider a straightforward extension to consider biophysically realistic post-synaptic potentials.

### Average and pre-spike mean release-site occupancy

The statistics of a binary occupancy variable *x*(*t*), where *x* = 1 if the site is occupied and is zero otherwise, are first considered. We first write down an obvious steady-state result which links two averages of this quantity: 〈*x*〉, the occupancy averaged over time, and 〈*x*〉_∞_, the mean occupancy just before the arrival of a presynaptic spike. It should be noted that these two quantities are only the same for (memory-less) Poisson processes. Given that the total restock rate must equal the total release rate in the steady state, the following balance equation holds

$$\begin{array}{c} \lambda \left(1-\left\langle x\right\rangle \right)=pr{\left\langle x\right\rangle }_{\infty } \end{array}$$ (5)

where λ is the restock rate given no vesicle is present, *p* is the probability of release given a vesicle is present and *r* is the firing rate of the presynaptic neuron. As will be seen later, it is the quantity 〈*x*〉_∞_ that is required for analysing the effect on the post-synaptic cell.

To derive 〈*x*〉_∞_ it is convenient to first consider a less complete expectation of *x*, denoted by $\bar{x}$, which implies the expectation of *x* for a fixed pattern of spike times {*t*_1_, *t*_2_, …*t*_*m*_} but averaged over all possible patterns of restock and release events. Consider the expected occupancy ${\bar{x}}_{m}$ immediately before the *m*th spike at time *t*_*m*_ as a function of the expected occupancy ${\bar{x}}_{m-1}$ immediately before the (*m* − 1)th spike: this obeys the recursion equation

$$\begin{array}{ccc} {\bar{x}}_{m} & = & {\bar{x}}_{m-1}qe^{-\lambda (t_{m}-t_{m-1})}+\left(1-e^{-\lambda (t_{m}-t_{m-1})}\right) \end{array}$$ (6)

where *q* = 1 − *p*. This can be solved, with the initial condition ${\bar{x}}_{1}=1$, to give

$$\begin{array}{c} {\bar{x}}_{m}=1-\frac{p}{q}\sum_{k=1}^{m-1}q^{k}e^{-\lambda (t_{m}-t_{m-k})}. \end{array}$$ (7)

Taking expectations over all realisations of presynaptic spike times, as *m* → ∞, gives the expected occupancy before the arrival of a presynaptic action potential in the steady-state

$$\begin{array}{c} {\left\langle x\right\rangle }_{\infty }=1-\frac{p}{q}\sum_{k=1}^{\infty }q^{k}\left\langle e^{-\lambda T_{k}}\right\rangle \end{array}$$ (8)

where *T*_*k*_ is the sum of the last *k* ISIs. This result is quite general and holds for arbitrarily correlated spike trains; however, we now consider the specific case of spike-times generated by a renewal process. In this case the ISIs will be independent so that the expectation over the exponential term factorises $\langle e^{-\lambda T_{k}}\rangle ={\langle e^{-\lambda t}\rangle }^{k}$ and the sum can be evaluated for 〈*x*〉_∞_ to give

$$\begin{array}{ccc} {\left\langle x\right\rangle }_{\infty }=\frac{1-\langle e^{-\lambda t}\rangle }{1-q\langle e^{-\lambda t}\rangle } & \;\text{and}\; & \left\langle x\right\rangle =1-p\frac{r(1-\left\langle e^{-\lambda t}\right\rangle )}{\lambda (1-q\left\langle e^{-\lambda t}\right\rangle )}. \end{array}$$ (9)

The corresponding form for 〈*x*〉 was found from Eq (5). The expectation 〈*e*^−λ*t*^〉 is straightforward to evaluate for many classes of renewal process and is directly related to the ISI Laplace or Fourier transform (Eqs 1 and 2) $L\left(\lambda \right)=\langle e^{-\lambda t}\rangle$.

#### Release-site occupancy for gamma-distributed ISIs

**Fig 1:**
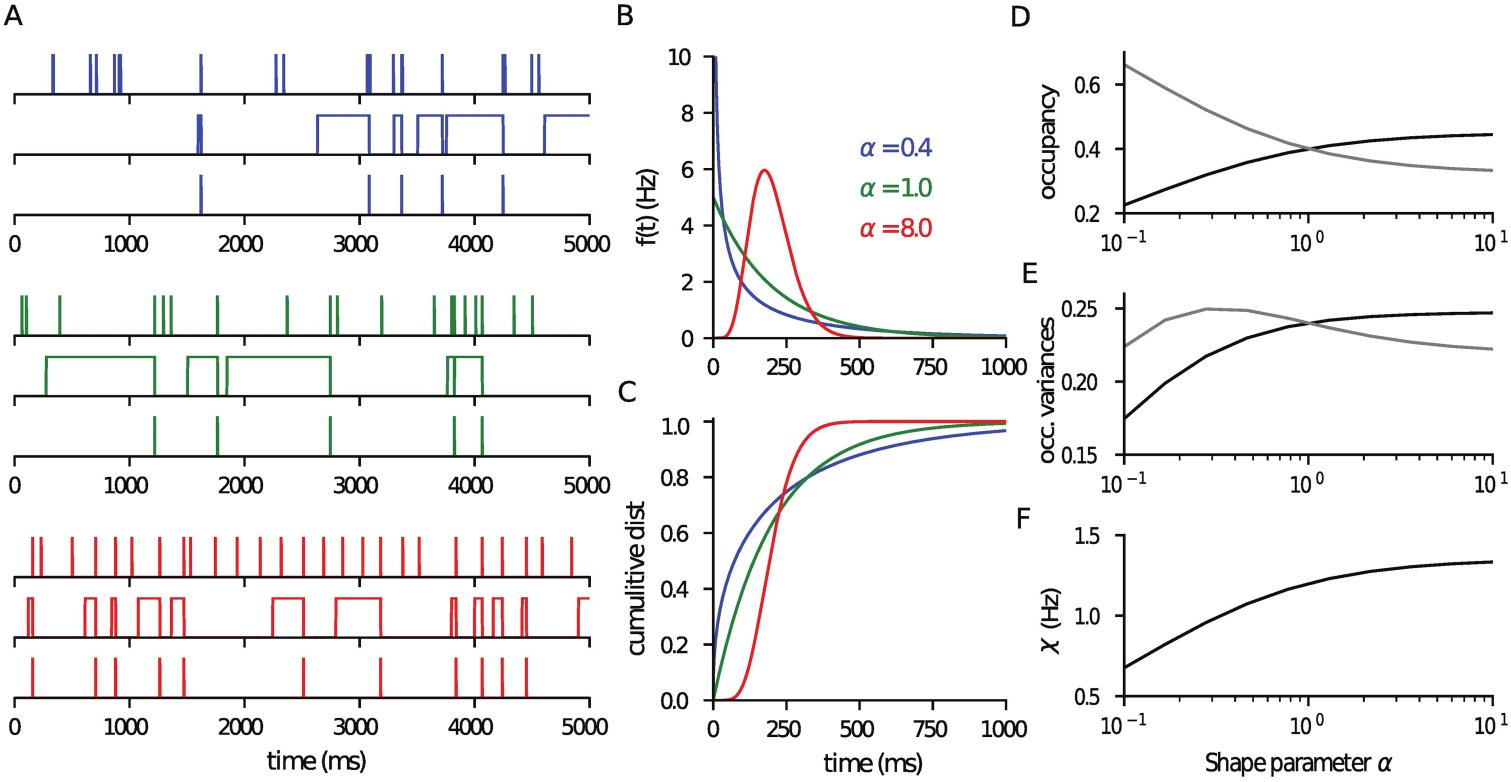
Vesicle occupancy and neurotransmitter release rate are a function of the ISI distribution. (A) Presynaptic spike train, vesicle occupancy and neurotransmitter release time-course for gamma-distributed ISIs with *α* as marked (in panel B) for the same release probabilty *p* = 0.6, presynaptic rate *r* = 5Hz and postsynaptic rate λ = 2Hz. (B) Corresponding ISI distributions for the three *α* values. (C) The cumulative distribution. (D) The mean 〈*x*〉 (grey) and the pre-spike 〈*x*〉_∞_ (black) release-site occupancies (Eqs 9 and 10). Note that 〈*x*〉_∞_ increases with increasing regularity, larger *α*. (E) Variance of the two occupancies exhibiting non-monotonic behaviour. (F) The mean neurotransmitter release rate 〈*χ*〉 is directly proportional to 〈*x*〉_∞_ and so shares its qualitative dependence on *α*. The code used to generate this figure is provided in the Supporting Material.

To illustrate the theoretical results in this paper we consider ISIs that have a distribution given by a gamma function with shape factor *α* and mean rate *r* (Eq 3). This is a convenient choice because a value *α* < 1 generates a spike train that is bursty, *α* = 1 is Poissonian firing and *α* > 1 is regular firing, thereby allowing for a range of behaviours to be examined as a single parameter is varied (at fixed presynaptic rate). Illustrations of the behaviour for three different values of *α*, from bursting to regular, are provided in Fig 1A with the corresponding ISI distribution (Eq 3) and its cumulation given in Fig 1B and 1C. For this class of ISI distribution we have

$$\begin{array}{c} \left\langle e^{-\lambda t}\right\rangle ={\left(\frac{\alpha r}{\alpha r+\lambda }\right)}^{\alpha } \end{array}$$ (10)

from Eq (4). This result can be used to calculate the forms of the average occupancy and mean pre-spike occupancy given in equation pair (9) and plotted as a function of *α* in Fig 1D. As *α* increases, the pre-spike mean 〈*x*〉_∞_ increases and the overall mean 〈*x*〉 decreases. The two means take the same value when *α* = 1 and the input spike train is an uncorrelated Poisson process. It is interesting to consider the two extreme limits of the quantity in Eq (10)

$$\begin{array}{ccc} \left\langle e^{-\lambda t}\right\rangle \to 1-\alpha \log\left(\lambda /\alpha r\right)\;\text{when}\;\alpha \to 0 & \text{and} & \left\langle e^{-\lambda t}\right\rangle \to e^{-\frac{\lambda }{r}}\;\text{when}\;\alpha \to \infty \end{array}$$ (11)

for highly bursty and highly regular presynaptic firing, respectively. In the limit *α* → 0 of very bursty presynaptic firing the mean occupancies tend to

$$\begin{array}{ccc} {\left\langle x\right\rangle }_{\infty }\to \frac{\alpha }{p}\log(\lambda /\alpha r) & \;\text{and}\; & \left\langle x\right\rangle \to 1-\frac{r}{\lambda }\alpha \log(\lambda /\alpha r). \end{array}$$ (12)

Hence, in the bursty limit the dominant behaviour of 〈*x*〉_∞_ is as $-\frac{\alpha }{p}\log\left(\alpha \right)$, which is dependent on *p* and *α* only, and the probability of neurotransmitter release *p*〈*x*〉_∞_ is a function of *α* only. Both these quantities and the variance of the prespike occupancy vanish in the extreme bursty limit *α* → 0, a trend that can be seen in Fig 1D, 1E and 1F.

As the presynaptic firing becomes increasingly regular, for large *α*, the quantity 〈*e*^−λ*t*^〉 begins to lose its *α* dependency. For the particular case of regular firing when λ/*r* ≪ 1, meaning that the restock rate is low relative to the presynaptic firing rate and the vesicles are in a depleted state the limit is 〈*x*〉_∞_ → (1/*p*)(λ/*r*) and so in this case the probability of neurotransmitter release *p*〈*x*〉_∞_ → λ/*r* is independent of both *p* and *α* and that the total mean rate of vesicle release *rp*〈*x*〉_∞_ is a function of λ only. This observation, that when *r* ≫ λ, the release rate loses its dependency on the presynaptic rate has been previously highlighted [32] for Poissonian processes (when *α* = 1). However, it is interesting to note that for bursty firing the release rate does not lose its dependency on the presynaptic rate so readily in this limit, as argued above.

### Vesicle release average and autocovariance

We now consider the statistics of the release of neurotransmitter and, in particular, the autocovariance of the release. This quantity will be required to calculate the variance of the postsynaptic voltage. We define the release events at a single site as a series of Dirac-delta events

$$\begin{array}{c} \chi \left(t\right)=\sum_{\left\{t_{k}\right\}}\delta \left(t-t_{k}\right) \end{array}$$ (13)

where here {*t*_*k*_} are the times of the neurotransmitter release events. These occur with probability *p* only when a presynaptic spike arrives and a vesicle is present, so that the steady-state mean of this quantity is 〈*χ*〉 = *pr*〈*x*〉_∞_, as already observed in the previous section.

We now consider the steady-state autocovariance that, because of its time-translation invariance, can be written 〈*χ*(*t*)*χ*(0)〉 − 〈*χ*〉^2^. For *t* > 0 we note that 〈*χ*(*t*)*χ*(0)〉 = 〈*χ*(*t*|0)〉〈*χ*〉 where 〈*χ*(*t*|0)〉 is understood to be the probability density of a vesicle being released at time *t* given that one was released previously at time 0. The autocovariance can therefore be written

$$\begin{array}{c} \mathrm{AutoCov}\left(\chi \right)=\left\langle \chi \right\rangle (\delta (t)+\langle \chi (t|0)\rangle -\langle \chi \rangle ) \end{array}$$ (14)

for *t* ≥ 0 (the function is even in time, thus specifying the *t* < 0 component) with the Dirac delta function coming from the zero-time contribution.

The quantity 〈*χ*(*t*|0)〉 itself is the rate of release, given a release at *t* = 0 and can be written as *pG*(*t*) where *G*(*t*) is the probability density of presynaptic spike arriving while a vesicle is present at time *t* given that initially (at *t* = 0) a presynaptic spike arrived and immediately after there was no vesicle present. We also introduce a related quantity *H*(*t*) which has the same conditionality but is the density of a presynaptic spike arriving while there is no vesicle present at a time *t*. Note that *F*(*t*) = *G*(*t*) + *H*(*t*) where *F*(*t*) is the probability density of an action potential arriving at *t* given there was one at *t* = 0 (the conditionality is the same because the arrival of a spike at t does not depend on whether a release site was stocked or not just before an earlier spike). This last quantity can be directly related to the ISI distribution *f*(*t*) via a convolution

$$\begin{array}{c} F\left(t\right)=f\left(t\right)+\int_{0}^{t}dsF\left(s\right)f\left(t-s\right) \end{array}$$ (15)

that can be solved in terms of integral transforms. It is straightforward to derive similar formulae for *G*(*t*) and *H*(*t*) via the introduction of the quantities

$$\begin{array}{ccc} g\left(t\right)=f\left(t\right)\left(1-e^{-\lambda t}\right) & \;\text{and}\; & h\left(t\right)=f\left(t\right)e^{-\lambda t} \end{array}$$ (16)

where *g*(*t*) and *h*(*t*) are the probability densities that the release site (initially empty at *t* = 0 following a presynaptic spike) are either restocked or not, respectively, by the time the next spike arrives; note also that *f*(*t*) = *g*(*t*) + *h*(*t*). To derive a self-consistent integral equation for *G*(*t*) we need to decompose it into the various histories that start with a vesicle absent at *t* = 0 following a presynaptic spike and end with a vesicle present at *t* when a spike arrives. There are four distinct contributions that need to be accounted for. The first is straightforward as it arises from the first spike arriving at *t* giving a contribution of *g*(*t*). The other contributions imply that the penultimate spike arrives at an intermediate time *s* that needs to be integrated over (as in Eq 15). The second contribution has a vesicle present before the intermediate time *s* and no release and so contributes *G*(*s*)*qf*(*t* − *s*). The third contribution has a vesicle present before the intermediate time *s* and there is a release and so contributes *G*(*s*)*pg*(*t* − *s*). The final contribution has no vesicle present before the intermediate time *s* and then there is a restock, contributing *H*(*s*)*g*(*t* − *s*). Combining these four contributions and simplifying, using *H* = *F* − *G* and *h* = *f* − *g*, results in the following integral equation

$$\begin{array}{c} G\left(t\right)=g\left(t\right)+\int_{0}^{t}dsF\left(s\right)g\left(t-s\right)+q\int_{0}^{t}dsG\left(s\right)h\left(t-s\right). \end{array}$$ (17)

The equations for *F*(*t*) and *G*(*t*) can either be solved numerically using iterative procedures or, alternatively, solved using integral transforms (see the next section). Noting that in the limit *t* → ∞ the conditional quantity *G*(*t*) converges to *r*〈*x*〉_∞_ allows the autocovariance

$$\begin{array}{c} \mathrm{AutoCov}\left(\chi \left(t\right)\right)=pr{\left\langle x\right\rangle }_{\infty }(\delta \left(t\right)+p(G\left(|t|\right)-r{\left\langle x\right\rangle }_{\infty })) \end{array}$$ (18)

of the neurotransmitter release time series *χ*(*t*) to be written in terms of *G*(*t*) and the occupancy 〈*x*〉_∞_.

#### Vesicle-release train in the Laplace domain and its power spectrum

As will be seen later, the Laplace transform of the densities *F*(*t*) and *G*(*t*) are required for the calculation of the post-synaptic voltage variance. The Laplace transforms of *g*(*t*) and *h*(*t*) can be written in terms of the ISI-distribution Laplace transform

$$\begin{array}{ccc} L_{g}\left(z\right)=L\left(z\right)-L\left(z+\lambda \right) & \;\text{and}\; & L_{h}\left(z\right)=L\left(z+\lambda \right). \end{array}$$ (19)

With these results and the convolution theorem, it is straightforward to solve the integral Eqs (15) and (17) in the Laplace domain.

$$\begin{array}{ccc} L_{F}\left(z\right)=\frac{L(z)}{1-L(z)} & \;\text{and}\; & L_{G}\left(z\right)=\frac{1}{\left(1-L(z)\right)}\;\frac{L(z)\;-\;L(z+\lambda )}{\left(1-qL(z+\lambda )\right)}. \end{array}$$ (20)

These results will be of use for the later calculation of the voltage variance. It is also useful to state the solution in the Fourier domain, as this is required for the power spectra of the spike train and the release process. Because both *F*(*t*) and *G*(*t*) tend to a constant as *t* → ∞ and so the Fourier transform will diverge at zero frequency, we separate the solutions into non-zero and zero frequency components to give the Fourier transforms as $\hat{F}\left(\omega \right)=L_{F}\left(i\omega \right)+r\pi \delta \left(\omega \right)$ and $\hat{G}\left(\omega \right)=L_{G}\left(i\omega \right)+r{\langle x\rangle }_{\infty }\pi \delta \left(\omega \right)$. These results can be used [40] to extend the autocorrelation of reference [29] to account for the biophysically important case of unreliable vesicle release. The power spectrum [40] of the presynaptic action potentials ${\hat{S}}_{\varrho }$ as well as that for the release events ${\hat{S}}_{\chi }$ follows as

$$\begin{array}{ccc} {\hat{S}}_{\varrho }\left(\omega \right)=r(1+2ℜL_{F}\left(i\omega \right)) & \;\text{and}\; & {\hat{S}}_{\chi }\left(\omega \right)=\left\langle \chi \right\rangle (1+2pℜL_{G}\left(i\omega \right))) \end{array}$$ (21)

where 〈*χ*〉 = *pr*〈*x*〉_∞_.

#### Auto-covariance and power spectrum for gamma-distributed ISIs

**Fig 2:**
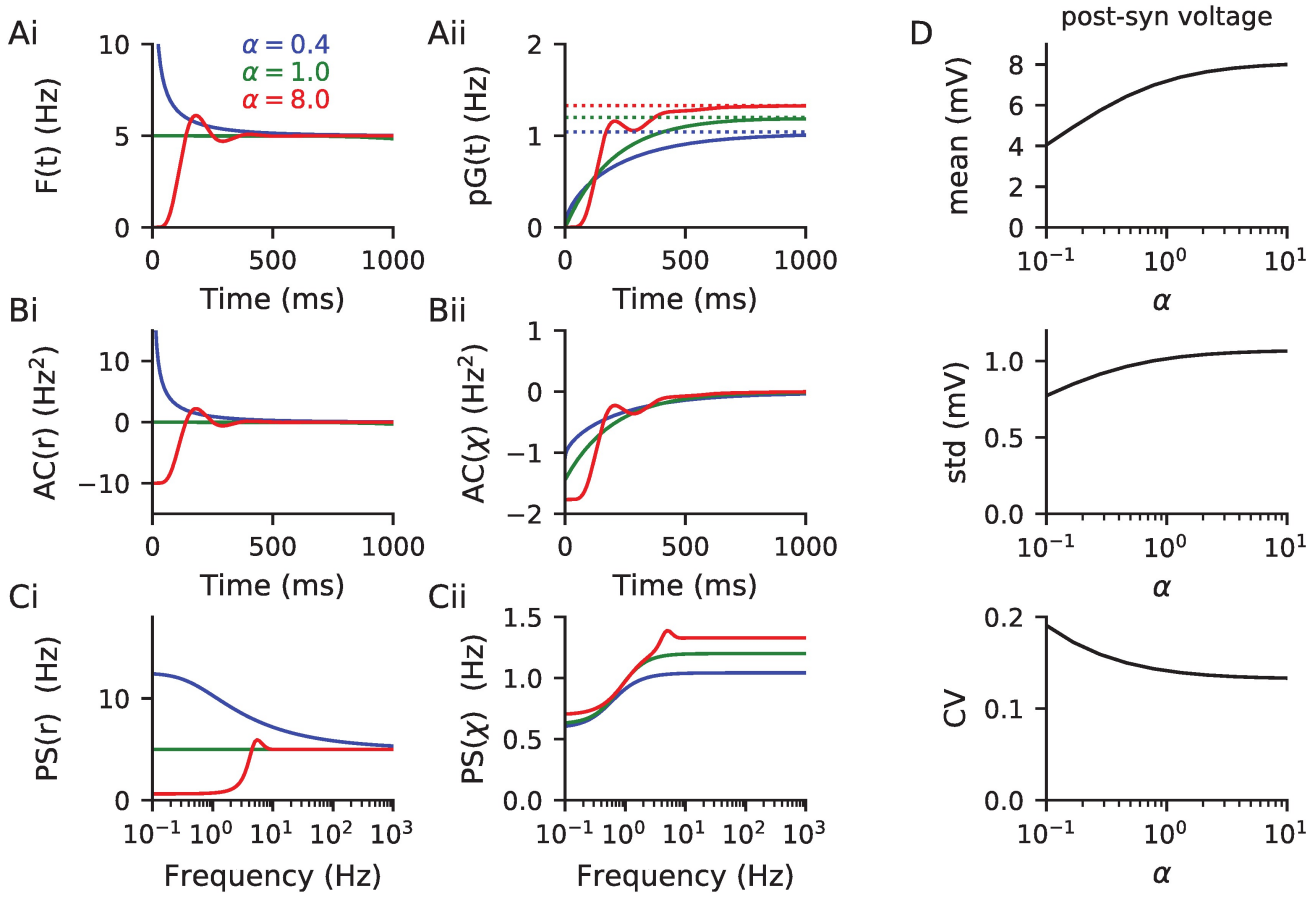
Temporal and spectral statistics of the presynaptic spike-train and neurotransmitter release required for post-synaptic voltage mean and variance. (A) The spike-train (Ai) and neurotransmitter release (Aii) event-triggered rate for three cases ranging from bursty to regular with *α* as marked (see Eq 22 and surrounding text). (B) The equivalent autocovariances of the spike train (Bi) and synaptic release events (Bii). For bursty presynaptic trains (small *α* = 0.4) the autocovariance is positive; however all cases have negative autocovariances for the release events themselves (Eq 18) because of the filtering by the depressing synapses. (C) The corresponding power spectra (Eq 21) of the spike train (Ci) and synaptic release events (Cii). (D) The postsynaptic voltage mean, standard deviation (std) and coefficient of variation std/mean (Eqs 24 and 29). Though the mean and std both increase with increasing presynaptic regularity, mirroring the behaviour of 〈*x*〉_∞_ (Fig 1D), the voltage CV itself decreases with increasing regularity. For panels A-C parameters and color are same as Fig 1, and in panel D parameter *α* is varied with other parameters provided in Table 1. The code used to generate this figure is provided in the Supporting Material.

For the particular case of gamma-distributed ISIs, the integral equation (Eq 15) for *F*(*t*) can be found by iteratively substituting for *F*(*s*) under the integral to provide a series of multiple integrals over products of *f*(*s*). On substituting for the gamma-distribution form for *f*(*t*), and using standard results for the normalisation of Dirichlet distributions, the following series solution is found

$$\begin{array}{c} F\left(t\right)=\frac{e^{-\alpha rt}}{t}\sum_{m=1}^{\infty }\frac{{\left(\alpha rt\right)}^{m\alpha }}{\Gamma (m\alpha )} \end{array}$$ (22)

which converges relatively rapidly. We were unable to find a similarly compact series solution for *G*(*t*), given in Eq (17); however, it is straightforward to develop a numerical scheme that solves the integral equation by iterating forward in time on a grid of time points. Fig 2A plots the presynaptic spike-triggered average rate *F*(*t*) together with the corresponding release-triggered average rate *pG*(*t*) for three choices of *α* ranging from bursty to regular (the same as those used in Fig 1). The late-time asymptote for *F*(*t*) is the presynaptic rate whereas for *pG*(*t*) it is the average release rate 〈*χ*〉. The corresponding finite component of the autocovariance for these processes is plotted in Fig 2B for positive time (they are even in time). Note that though the presynaptic rate is positively correlated for bursty spike trains, its autocovariance becomes negative at short times (like for the Poissonian and regular trains) once it has passed through synapses exhibiting short-term depression.

### The postsynaptic response

In the previous section the pre-spike occupancy of a release site was calculated and the release-train autocovariance derived. We now use these results to derive the voltage mean and variance of the postsynaptic neuron. The subthreshold mean and variance of the postsynaptic neuron are the key quantities necessary to estimate the output firing rate of a neuron [18, 36, 41–45] and hence its resultant effect on the network.

It is assumed that the presynaptic neurons are uncorrelated and each fires at a rate *r* with the same ISI distribution. For simplicity we assume that each neurotransmitter-release event causes the postsynaptic membrane to increase by a fixed voltage *a* (this restriction is for simplicity and can be relaxed). In this section we consider presynaptic cells that make contacts with only one vesicle release site, leaving multiple release sites to a later section. The postsynaptic voltage therefore follows the equation

$$\begin{array}{c} \tau \frac{dv}{dt}=\mu -v+a\tau \sum_{i=1}^{N}\chi_{i}\left(t\right) \end{array}$$ (23)

where *τ* is the membrane time constant, *μ* the resting potential in absence of synaptic drive and there are *N* presynaptic neurons, with the *i*th having a release train *χ*_*i*_(*t*).

The steady-state mean post-synaptic voltage is found using the result 〈*χ*〉 = *pr*〈*x*〉_∞_ so that

$$\begin{array}{c} \left\langle v\right\rangle =\mu +a\tau Npr{\left\langle x\right\rangle }_{\infty }. \end{array}$$ (24)

This is an increasing function of the prespike occupancy 〈*x*〉_∞_ and therefore also increases with the regularity of the presynaptic spike train (see Fig 1D for the dependence of 〈*x*〉_∞_ on the burstiness of the presynaptic spike train).

To find the post-synaptic voltage variance we first solve differential Eq (23) formally as an integral over the release trains

$$\begin{array}{c} v\left(t\right)=\left\langle v\right\rangle +a\int_{-\infty }^{t}dt^{\prime }e^{-(t-t^{\prime })/\tau }\sum_{i}(\chi_{i}\left(t^{\prime }\right)-\left\langle \chi \right\rangle ) \end{array}$$ (25)

where we included the component of the mean stemming from the synaptic input in the summation. The voltage variance can therefore be written as a double integral over the autocovariance of *χ*(*t*) as follows

$$\begin{array}{c} \mathrm{Var}\left(v\right)=Na^{2}\left\langle \chi \right\rangle \int_{0}^{\infty }ds\int_{0}^{\infty }ds^{\prime }e^{-s/\tau }e^{-s^{\prime }/\tau }(\delta \left(s\;-\;s^{\prime }\right)+p(G(|s\;-\;s^{\prime }\left|\right)-r{\left\langle x\right\rangle }_{\infty })) \end{array}$$ (26)

where the fact that the *χ*_*i*_(*t*) from different presynaptic neurons are uncorrelated has been used. The Dirac-delta component is straightforward to evaluate and the other components are symmetric around *s* − *s*′ so that

$$\begin{array}{c} \mathrm{Var}\left(v\right)=\frac{\tau Na^{2}}{2}\left\langle \chi \right\rangle +2Na^{2}\left\langle \chi \right\rangle p\int_{0}^{\infty }ds^{\prime }e^{-s^{\prime }/\tau }\;\int_{s^{\prime }}^{\infty }dse^{-s/\tau }(G(|s-s^{\prime }\left|\right)-r{\left\langle x\right\rangle }_{\infty }) \end{array}$$ (27)

which, on a change of the inner integration variable *z* = *s* − *s*′, allows for the outer integral over *s*′ to be performed resulting in

$$\begin{array}{c} \mathrm{Var}\left(v\right)=\frac{\tau Na^{2}}{2}\left\langle \chi \right\rangle (1+2p\int_{0}^{\infty }dze^{-z/\tau }(G\left(z\right)-r{\left\langle x\right\rangle }_{\infty })). \end{array}$$ (28)

Part of the integral in the above equation is simply the Laplace transform of *G*(*t*), which was already provided in the second of equation pair (20). On substitution, this gives a compact form for the postsynaptic voltage variance in terms of $L\left(z\right)=\langle e^{-zt}\rangle$ the Laplace transform of the ISI distribution

$$\begin{array}{c} \mathrm{Var}\left(v\right)=\frac{\tau Na^{2}}{2}\left\langle \chi \right\rangle (1+2p(\frac{L(1/\tau )-L(1/\tau +\lambda )}{\left(1-L(1/\tau ))(1-qL(1/\tau +\lambda )\right)}-\tau r{\left\langle x\right\rangle }_{\infty })). \end{array}$$ (29)

This is a central result of this paper and is general for neurons that receive synapses with single release sites from presynaptic neurons that fire as a renewal process. We now go on to examine the postsynaptic voltage behaviour for gamma-distributed presynaptic ISIs, and consider the case for presynaptic integrate-and-fire models in a later subsection.

#### Post-synaptic voltage statistics for gamma-distributed ISIs

The expectations appearing in the voltage mean and variance equation are of the form of Eq (4) for the case of gamma-distributed ISIs. The mean post-synaptic voltage is a linear function of 〈*x*〉_∞_ and therefore also of 〈*χ*〉 (Fig 1D and 1F), which in turn take their *α* dependence from Eq (10). The voltage mean therefore increases monotonically with increasing regularity of the presynaptic train, as can be seen in Fig 2D. For the calculation of the variance (Eq 29) two additional expectations are required

$$\begin{array}{ccc} L(1/\tau )\; & \;=\; & \;\left\langle e^{-t/\tau }\right\rangle ={\left(\frac{\alpha \tau r}{1+\alpha \tau r}\right)}^{\alpha }\;\text{and}\;\\ L(1/\tau +\lambda )\; & \;=\; & \;\left\langle e^{-(1/\tau +R_{r})T}\right\rangle ={\left(\frac{\alpha \tau r}{1+\tau \lambda +\alpha \tau r}\right)}^{\alpha }. \end{array}$$ (30)

Using these results the voltage standard-deviation (std) is plotted in Fig 2D along with the coefficient of variation (CV) of the post-synaptic voltage (std/mean). While the variance increase with increasing regularity of the presynaptic train passing through depressing synapses, it can be noted that the voltage CV itself decreases (last panel of Fig 2D).

### Multiple release sites sharing the same presynaptic neuron

**Fig 3:**
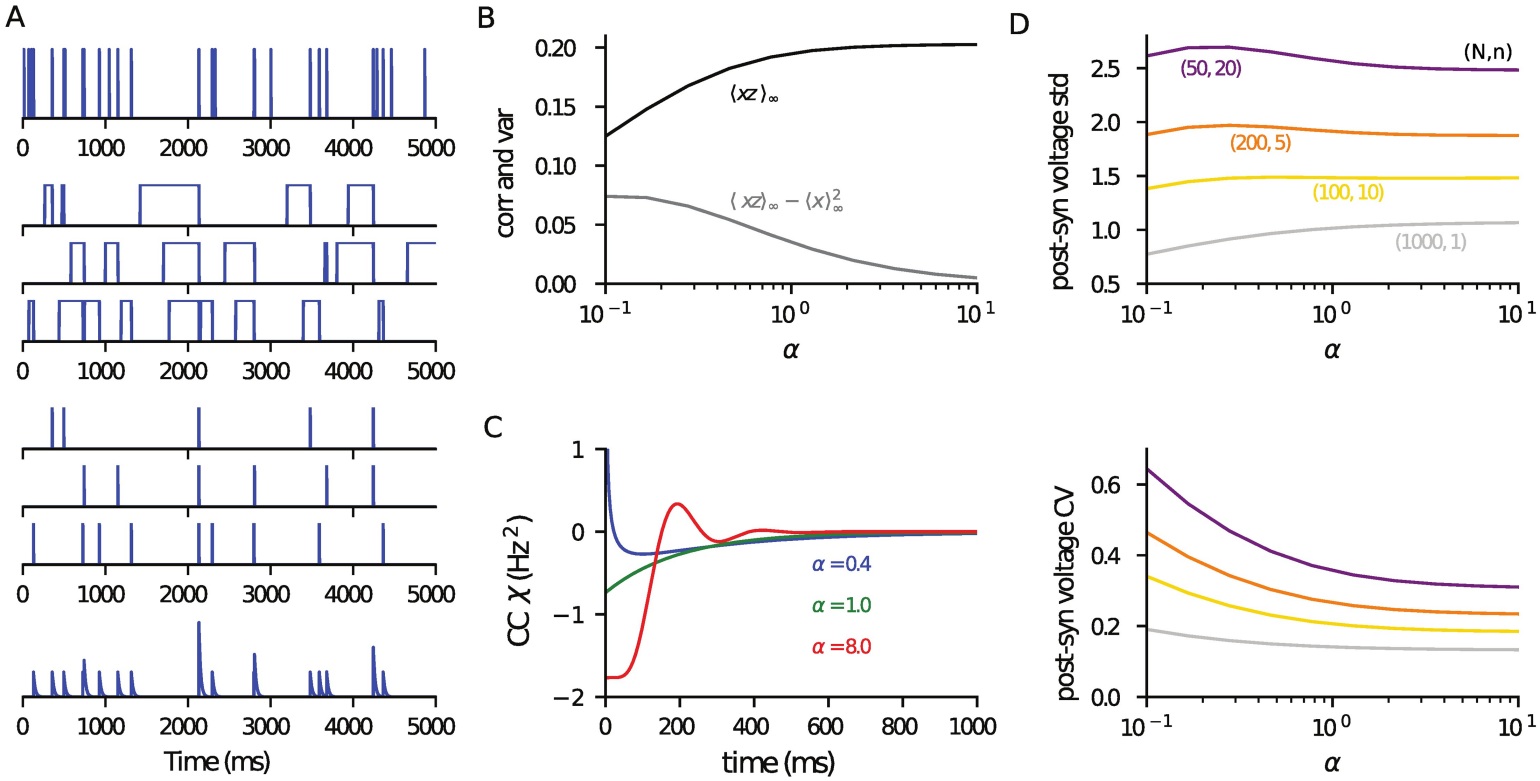
Correlated occupancy, release and post-synaptic voltage statistics when there are multiple contacts per presynaptic cell. (A) Illustration of a case with one presynaptic cell *N* = 1 making three contacts *n* = 3. Shown in descending order: presynaptic spike train (*α* = 0.4, *r* = 5Hz), occupancy of the three release sites (restock λ = 2Hz), the corresponding release-event time courses (*p* = 0.6), and the voltage time course (see Table 1 for parameters). (B) The occupancy correlation 〈*xz*〉_∞_ for a pair of sites receiving the same presynaptic spike train and the covariance ${\langle xz\rangle }_{\infty }-{\langle x\rangle }_{\infty }^{2}$ (see Eqs 34 and 35). (C) The cross-covariance of release events from a pair of sites sharing the same presynaptic cell (Eq 39). (D) Post-synaptic voltage standard deviation (top: from Eq 42) and CV (bottom) as a function of *α* for four combinations of *N*, *n* as marked. With each of these choices, where *Nn* = 1000, the mean voltage (Eq 32) is the same as in Fig 2D. The code used to generate this figure is provided in the Supporting Material.

A single presynaptic neuron will typically make contacts with a postsynaptic cell that result in multiple vesicle release sites, with estimates of this parameter varying from 1 to as much as 100 [46] for neocortical layer-5 pyramidal cells. Here we consider a case where each of the *N* presynaptic cells makes connections with *n* indepedent release sites, a scenario illustrated in Fig 3A for a case *N* = 1 and *n* = 3. We assume that all processes (such as restock and release) are statistically independent at each of these sites, but those sharing the same presynaptic neuron receive the same spike train. The postsynaptic-voltage dynamics now take the form

$$\begin{array}{c} \tau \frac{dv}{dt}=\mu -v+a\tau \sum_{i=1}^{N}\sum_{j=1}^{n}\chi_{ij}\left(t\right) \end{array}$$ (31)

where here *χ*_*ij*_ is the release time course of the *j*th contact on the *i*th neuron. Only the *χ*_*ij*_ that share the same presynaptic neuron will be correlated. The steady-state mean voltage is straightforward to derive using the result 〈*χ*〉 = *pr*〈*x*〉_∞_ so that

$$\begin{array}{c} \left\langle v\right\rangle =\mu +a\tau Nnpr{\left\langle x\right\rangle }_{\infty }. \end{array}$$ (32)

However, to calculate the variance correlations between release sites need to be accounted for, which requires the solution of an additional integral equation.

#### Expectation of joint vesicle occupancy for release sites

The *j*th contact of presynaptic cell *i* will have a release train *χ*_*ij*_(*t*) that is correlated with that of other contacts from the same presynaptic cell due to receiving the same spike train. To take this into account, the first quantity to consider is the joint prespike occupancy 〈*xz*〉_∞_ for two release sites (labelled *x*(*t*) and *z*(*t*)) sharing the same presynaptic cell. Given that restock and release events are uncorrelated, and that only the spike times are, it is possible to simply multiply the result Eq (6) by the equivalent for ${\bar{z}}_{m}$ and take expectations

$$\begin{array}{c} {\left\langle xz\right\rangle }_{m}={\left\langle xz\right\rangle }_{m-1}q^{2}\left\langle e^{-2\lambda t}\right\rangle +2{\left\langle x\right\rangle }_{m-1}q\left\langle e^{-\lambda t}\left(1-e^{-\lambda t}\right)\right\rangle +\left\langle {\left(1-e^{-\lambda t}\right)}^{2}\right\rangle \end{array}$$ (33)

where it should be noted that first-order expectations 〈*x*〉_*m*_ = 〈*z*〉_*m*_ are identical for the two release sites as they are statistically indistinguishable. In the steady-state limit *m* → ∞ the difference Eq (33) results in

$$\begin{array}{c} {\left\langle xz\right\rangle }_{\infty }=\frac{2q{\left\langle x\right\rangle }_{\infty }\left\langle \left(1-e^{-\lambda t}\right)e^{-\lambda t}\right\rangle +\left\langle {\left(1-e^{-\lambda t}\right)}^{2}\right\rangle }{1-q^{2}\left\langle e^{-2\lambda t}\right\rangle } \end{array}$$ (34)

which gives the covariance as

$$\begin{array}{c} {\left\langle xz\right\rangle }_{\infty }-{\left\langle x\right\rangle }_{\infty }^{2}=p^{2}\frac{\left\langle e^{-2\lambda t}\right\rangle -{\left\langle e^{-\lambda t}\right\rangle }^{2}}{\left(1-q^{2}\left\langle e^{-2\lambda t}\right\rangle \right){\left(1-q\left\langle e^{-\lambda t}\right\rangle \right)}^{2}} \end{array}$$ (35)

where the expectations are Laplace transforms of the ISI distribution $\langle e^{-m\lambda t}\rangle =L\left(m\lambda \right)$. It will be necessary in the next section to find the steady-state probability that site *z* is still stocked given a vesicle was just released from site *x* and also the probability that site *z* is unstocked with the same conditionality. These probabilities are

$$\begin{array}{ccc} q{\left\langle xz\right\rangle }_{\infty }/{\left\langle x\right\rangle }_{\infty } & \;\text{and}\; & 1-q{\left\langle xz\right\rangle }_{\infty }/{\left\langle x\right\rangle }_{\infty } \end{array}$$ (36)

respectively.

#### Cross-covariance function for release sites

To compute the cross-covariance for two release sites sharing the same presynaptic cell requires the introduction of a quantity *G*′(*t*), which is the probability density that a spike arrives while a vesicle is present at time *t* given that the site was occupied immediately after a spike at time 0. Note that this differs from *G*(*t*) defined earlier only by the initial condition on the occupancy of a vesicle release site. In analogy, a quantity *H*′(*t*) can also be introduced which has the conditionality as *G*′(*t*) but is the probability density that a spike arrives while a vesicle is absent. Note that *F*(*t*) = *G*′(*t*) + *H*′(*t*). To derive an integral equation for *G*′(*t*), involving the previously defined quantities *f*(*t*), *g*(*t*) and *h*(*t*), we consider the four distinct histories that contribute to it. The first involves no intermediate spike between 0 and *t*, with the first spike arriving at *t*, and is therefore simply *f*(*t*). The other three contributions involve at least one intermediate spike, the penultimate one arriving at *s* and being integrated over. The first of these three contributions involves the penultimate spike arriving at a time *s* when a vesicle is present and there being no release, to give *G*′(*s*)*qf*(*t* − *s*). The second involves the penultimate spike arriving at a time *s* when a vesicle is present, there being a release and then the empty site being restocked, thereby contributing *G*′(*s*)*pg*(*t* − *s*). The final contribution involves there being no vesicle present at the penultimate spike time *s* but then being restocked, to give *H*′(*s*)*g*(*t* − *s*). Combining these four terms and then simplifying results in the following integral equation

$$\begin{array}{c} G^{\prime }\left(t\right)=f\left(t\right)+\int_{0}^{t}dsF\left(s\right)g\left(t-s\right)+q\int_{0}^{t}dsG^{\prime }\left(s\right)h\left(t-s\right). \end{array}$$ (37)

It will be seen later that this quantity typically only appears with *G*(*t*) subtracted from it. Eq (17) includes one of the integrals seen in *G*′(*t*) and hence the difference of these quantites obeys

$$\begin{array}{c} G^{\prime }\left(t\right)-G\left(t\right)=h\left(t\right)+q\int_{0}^{t}ds(G^{\prime }\left(s\right)-G\left(s\right))h\left(t-s\right) \end{array}$$ (38)

which is an integral equation with a similar structure to Eq (15) for the spike-triggered presynaptic rate *F*(*t*) and is simpler to treat analytically and numerically. The cross-covariance in vesicle release is a sum of *G*′(*t*) and *G*(*t*) weighted by the probabilities that the second site is stocked or unstocked immediately after the first releases (these are given in Eq 36) so that the cross-covariance can ultimately be written in the form

$$\begin{array}{c} \mathrm{CrossCov}\left(\chi_{x},\chi_{z}\right)\;=\;p^{2}r{\left\langle xz\right\rangle }_{\infty }\;(\delta \left(t\right)\;+\;\frac{{\left\langle x\right\rangle }_{\infty }}{{\left\langle xz\right\rangle }_{\infty }}\;(G(|t|)\;-\;r{\left\langle x\right\rangle }_{\infty })\;+\;q(G^{\prime }\;\left(|t|\right)\;-\;G(|t|))\;)\; \end{array}$$ (39)

where release trains *χ*_*x*_ and *χ*_*z*_ are from two distinct release sites that share the same presynaptic cell. To derive the post-synaptic voltage variance the Laplace transform of the difference *G*′(*t*) − *G*(*t*) is required. From Eq (38) this is

$$\begin{array}{ccc} \;L_{G^{\prime }}\left(z\right)-L_{G}\left(z\right)=\frac{L(z+\lambda )}{1-qL(z+\lambda )} & \text{so} & L_{G^{\prime }}\left(z\right)=\frac{L(z)(1-L(z+\lambda )}{\left(1-L(z))(1-qL(z+\lambda )\right)}. \end{array}$$ (40)

For completeness, the Fourier transform is ${\hat{G}}^{\prime }\left(\omega \right)=L_{G^{\prime }}\left(i\omega \right)+r{\langle x\rangle }_{\infty }\pi \delta \left(\omega \right)$.

#### Postsynaptic voltage variance for multiple release sites

The voltage Eq (31) has a solution analgous to Eq (25) but with a double sum over the *N* presynaptic neurons and their *n* contacts. On squaring this and taking expectations there are two groups of non-zero terms amongst the (*Nn*)^2^ components of the double sum: *Nn* terms of the auto-covariance (Eq 18) and *Nn*(*n* − 1) terms of the cross-covariance (Eq 39). The double integral over the cross-covariance can be performed in the same way as for the auto-covariance, and when combined results in the following form for the post-synaptic voltage variance

$$\begin{array}{ccc} \; & & \mathrm{Var}\left(v\right)=Nn\frac{\tau a^{2}}{2}pr{\left\langle x\right\rangle }_{\infty }\;(1+2p(L_{G}\left(1/\tau \right)-\tau r{\left\langle x\right\rangle }_{\infty }\;))+\\ \; & & Nn\left(n\;-\;1\right)\frac{\tau a^{2}}{2}p^{2}r{\left\langle xx^{\prime }\right\rangle }_{\infty }\;(1\;+\;\frac{2{\left\langle x\right\rangle }_{\infty }}{{\left\langle xx^{\prime }\right\rangle }_{\infty }}\;(L_{G}\left(1/\tau \right)\;-\;\tau r{\left\langle x\right\rangle }_{\infty }\;)\;+\;2q\;(L_{G^{\prime }}\left(1/\tau \right)\;-\;L_{G}\left(1/\tau \right))\;)\;.\\ \; & \end{array}$$ (41)

The first line arises from the autocorrelation of *χ* and the second line from the cross-correlation between *χ* and *χ*′. This form can be simplified and the equations for $L_{G}\left(z\right)$ and $L_{G^{\prime }}\left(z\right)$ used to get

$$\begin{array}{ccc} \;\mathrm{Var}(v)\; & \;=\; & \;\frac{\tau a^{2}Nn}{2}\left\langle \chi \right\rangle \;(1+\;2pn(\frac{L(1/\tau )-L(1/\tau +\lambda )}{\left(1\;-\;L(1/\tau ))(1\;-\;qL(1/\tau +\lambda )\right)}-\tau r{\left\langle x\right\rangle }_{\infty })\;)\\ & \;+\; & \;\frac{\tau a^{2}Nn}{2}\left\langle \chi \right\rangle \left(n-1\right)p\frac{{\left\langle xx^{\prime }\right\rangle }_{\infty }}{{\left\langle x\right\rangle }_{\infty }}(1+\frac{2qL(1/\tau +\lambda )}{1-qL(1/\tau +\lambda )}). \end{array}$$ (42)

This formula represents the extension of the analytical forms for the voltage variance to include biophysical details such as: stochastic transmission, quantal effects, short-term depression, multiple contacts, all with non-Poissonian input.

#### Post-synaptic voltage for gamma-distributed ISIs with multiple contacts

Extending the calculation of the variance of the post-synaptic voltage for gamma-distributed ISIs to the case of multiple contacts is straightforward as it still requires only knowledge of the Laplace transform of the ISI distribution. The temporal form of the cross-covariance, however, requires *G*′(*t*) which is given by an integral equation that is awkard to solve numerically. However, the equation for the difference (Eq 38) is easier to analyse and it is the difference that is required for the cross-covariance of the release events between sites sharing a presynaptic neuron. This quantity can be solved in the form of a series in much the same was as was done for *F*(*t*) in Eq (22), giving in this case

$$\begin{array}{c} G^{\prime }\left(t\right)-G\left(t\right)=\frac{e^{-(\alpha r+\lambda )t}}{qt}\sum_{m=1}^{\infty }\frac{q^{m}{\left(\alpha rt\right)}^{m\alpha }}{\Gamma (m\alpha )} \end{array}$$ (43)

which also converges rapidly. Fig 3B shows the correlation (Eq 34) and covariance (Eq 35) between two release sites *x* and *z* that share the same presynaptic neuron: note the contrasting behaviour as *α* scans from bursty to regular at constant presynaptic rate. Fig 3C shows the cross-covariance (Eq 39) for the same three *α* values used in Fig 2Bii. In Fig 3D the standard-deviation of the voltage and its CV are plotted for three pairs of *N* and *n*, such that their product is *Nn* = 1000. For this choice the voltage mean is the same as in Fig 2D. Note that the standard deviations have a qualitatively different dependence on the regularity of the presynaptic train for different ratios of contact number and presynaptic neuron number: for many contacts the std is highest for bursting presynaptic cells because the effects of the large voltage deviations from multiple release events exceed the effect of mean lower release rate for smaller values of *α*.

### ISIs generated by presynaptic integrate-and-fire neurons

In previous sections analytical forms for the pre-spike occupation 〈*x*〉_∞_, autocovariance of the release-train *χ* and the resulting voltage moments were derived with results illustrated using gamma-distributed ISIs (Eq 3). Integrate-and-fire models, such as the Leaky, Quadratic or Exponential IF models driven by white noise that, following a spike, retain no memory of their previous state will generate spike trains with uncorrelated ISIs. In this case the ISI distribution is identical to the first-passage-time density of the steady-state dynamics. Because of this, all the general results derived thus far are applicable when the presynaptic population is comprised of integrate-and-fire neurons. For the LIF the Fourier transform of the first-passage-time density is available analytically, whereas for non-linear IF models like the QIF or EIF it can be straightforwardly obtained numerically. We now consider examples of these two cases.

#### Presynaptic leaky integrate-and-fire neurons

We first consider a presynaptic population comprised of LIF neurons that are each driven by an input that has a constant and (independent) fluctuating component. The voltage evolution obeys the equation

$$\begin{array}{c} \tau \dot{v}=\mu -v+\sigma \sqrt{2\tau }\xi \left(t\right) \end{array}$$ (44)

where *τ* is the membrane time constant, *μ* is the voltage mean and *σ* its standard deviation in the absence of a threshold, and *ξ*(*t*) is zero-mean 〈*ξ*(*t*)〉 = 0, delta-correlated Gaussian white noise such that 〈*ξ*(*t*)*ξ*(*t*′)〉 = *δ*(*t* − *t*′). With a threshold at *v*_th_ and reset at *v*_re_ the neuron will fire at an average rate *r*, which can be written as a single integral [41]

$$\begin{array}{c} \frac{1}{r\tau }=\int_{0}^{\infty }\frac{dy}{y}e^{-y^{2}/2}\left(e^{yy_{\mathrm{th}}}-e^{yy_{\mathrm{re}}}\right) \end{array}$$ (45)

where *y*_th_ = (*v*_th_ − *μ*)/*σ* with an analogous definition for *y*_re_. The Fourier-transform $\hat{f}\left(\omega \right)$ of the first-passage-time density, or ISI distribution has been derived in terms of cylinder functions [47] and can also be written as a ratio of integrals [42] of similar form to Eq (45)

$$\begin{array}{c} \hat{f}\left(\omega \right)=\frac{\int_{0}^{\infty }dy\;y^{i\omega \tau }\frac{d}{dy}[e^{yy_{\mathrm{re}}-y^{2}/2}]}{\int_{0}^{\infty }dy\;y^{i\omega \tau }\frac{d}{dy}[e^{yy_{\mathrm{th}}-y^{2}/2}]}. \end{array}$$ (46)

Using the relation (Eq 2) between the expectation of an exponential, and the Fourier and Laplace transform of the ISI distribution, the key quantity required for all the main analytical results derived in this paper is

$$\begin{array}{c} L\left(z\right)=\left\langle e^{-zt}\right\rangle =\frac{\int_{0}^{\infty }dy\;y^{\tau z-1}e^{-y^{2}/2}e^{yy_{\mathrm{re}}}}{\int_{0}^{\infty }dy\;y^{\tau z-1}e^{-y^{2}/2}e^{yy_{\mathrm{th}}}} \end{array}$$ (47)

for ISIs generated by LIF neurons (note that a partial integration step has been performed between Eqs 46 and 47). For example, the mean release-site occupation (Eq 9) just before the arrival of a presynaptic spike takes the form

$$\begin{array}{c} {\left\langle x\right\rangle }_{\infty }=\frac{\int_{0}^{\infty }dy\;y^{\tau \lambda -1}e^{-\frac{y^{2}}{2}}\left(e^{yy_{\mathrm{th}}}-e^{yy_{\mathrm{re}}}\right)}{\int_{0}^{\infty }dy\;y^{\tau \lambda -1}e^{-\frac{y^{2}}{2}}\left(e^{yy_{\mathrm{th}}}-qe^{yy_{\mathrm{re}}}\right)}. \end{array}$$ (48)

for presynaptic LIF neurons. Though not explicitly addressed in this paper, the corresponding formulae for LIF neurons driven by Poissonian shot-noise [42] take a similar form, with 〈*x*〉_∞_ again expressible as a ratio of integrals.

**Fig 4:**
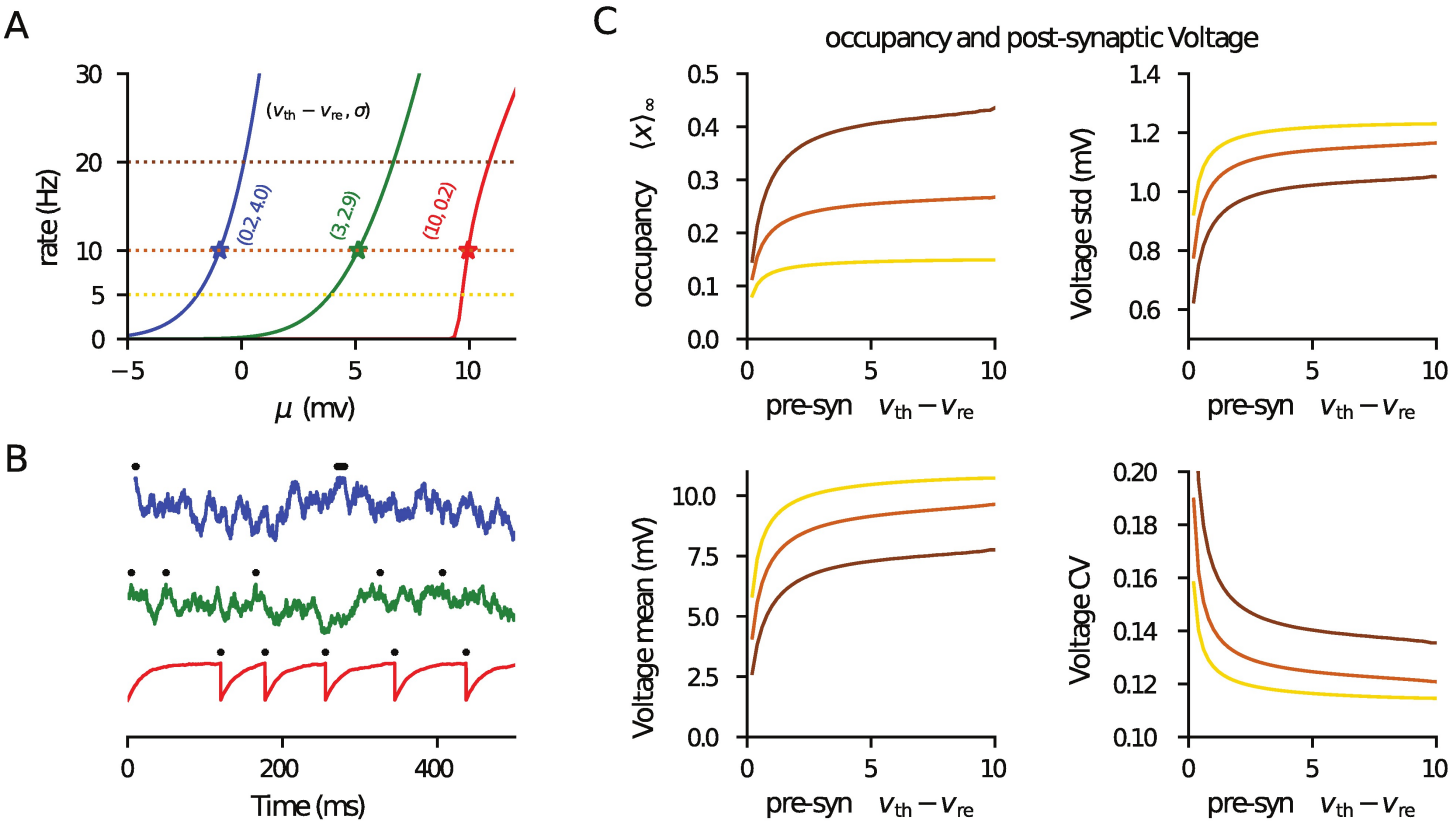
Occupancy and post-synaptic voltage statistics from a presynaptic pool of LIF neurons. (A) Presynaptic rate (Eq 45) as a function of drive *μ* for three pairs of (*v*_th_ − *v*_re_, *σ*) as marked leading to bursty (blue), intermediate (green) and regular (red) firing statistics. Lines of constant rate are at 5, 10 and 20Hz are marked (dotted lines). (B) Example presynaptic voltage time courses (Eq 44) with spikes marked (black) for the parameters marked with symbols in panel A (*μ* is varied so they are all at rate 5Hz). (C) The parameters *v*_re_ and *σ* were co-varied linearly (with *μ* compensating so that rates were constant at 5, 10 and 20Hz: see dotted lines in panel A, with same color coding used) and the occupancy (Eq 9), post-synaptic voltage mean (Eq 24), standard deviation (from Eq 29) and CV plotted (against *v*_th_ − *v*_re_). The behaviour seen is qualitatively the same as for the gamma-generated ISIs. In this figure *N* = 1000, *n* = 1 and *v*_th_ = 10mV, with other parameters given in Table 1. The code used to generate this figure is provided in the Supporting Material.

Fig 4 examines the release-site occupancy and post-synaptic voltage statistics when the presynaptic spike trains are generated by LIF models. At a fixed rate, a reset near threshold with strong noise will lead to bursting behaviour [43] whereas a low reset with weak noise noise will lead to regular spiking (varying the steady component *μ* allows the rate to be kept the same). In Fig 4A the presynaptic rates versus *μ* for three pairs of *v*_th_ − *v*_re_ and *σ* (values given in the figure) ranging from bursting (blue), intermediate (green) and regular (red) are plotted. Fig 4B shows example presynaptic-voltage time courses for these three cases when the presynaptic rate is 10Hz (symbols on Fig 4A). We can simultaneously vary *v*_re_ and *σ* linearly, from their values on the blue curve to those on the red curve (with *μ* compensating so that the rate is constant) to cover the range from bursty to regular firing. The resulting occupancy (Eq 9) and post-synaptic voltage mean, std and CV (Eqs 24 and 29) are plotted in Fig 4C and demonstrate qualitatively similar behaviour to that seen for gamma-distributed ISIs.

#### ISI distributions from exponential integrate-and-fire neurons

**Fig 5:**
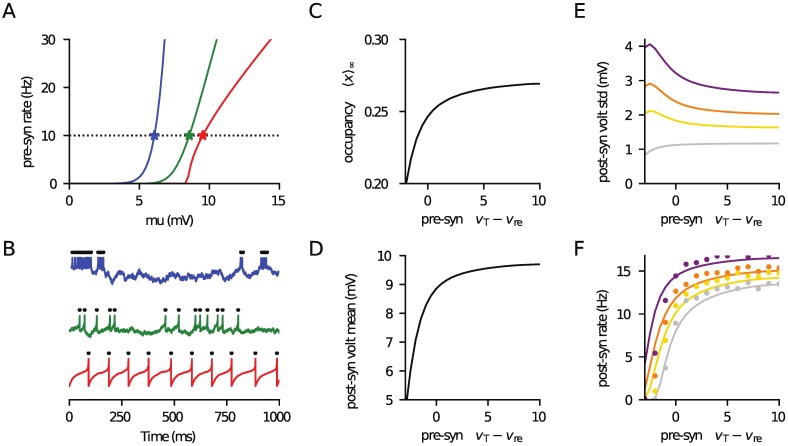
Approximate rate of a postsynaptic EIF neuron driven by a pool of presynaptic EIF neurons, using the matched-variance approximation. (A) Firing rate of presynaptic neurons as a function of *μ* for three pairs (*v*_T_ − *v*_re_, *σ*) representing bursting (−3mV, 2.0mV; blue), intermediate (1.0mV, 1.45mV; green) and regular (10mV, 0.2mV; red) firing. (B) Example presynaptic-voltage time courses (Eq 49) corresponding to symbols in panel A. In panels C-F parameters *v*_re_ and *σ* were simultaneously varied linearly between the values of the blue and red curves in panel A (*μ* adjusted so the presynaptic rate remained 10Hz). Hence, *v*_T_ − *v*_re_ ranges from bursting −3mV to regular 10mV. (C) Shows the mean pre-spike vesicle occupancy (Eq 9), (D) the mean post-synaptic voltage (Eq 32) and (E) its standard deviation (From Eq 42). In (F) the post-synaptic rate was approximated using the matched-variance approximation, in which the voltage mean and variance (exactly calculated for this filtered drive) are used in the white-noise EIF firing rate calculation. For comparison, the symbols are simulations of the true firing rate. In Panels D-F *Nn* = 1000 with (*N*, *n*) taking the values (1000, 1: grey), (100, 10: yellow), (50, 20: orange) and (25, 40: purple). The reset for the post-synaptic neuron was held constant at *v*_re_ = 5mV and other parameters are given in Table 1. The code used to generate this figure is provided in the Supporting Material.

A number of extensions of the LIF neuron model have been proposed to better capture the non-linearities at the onset of the spike. These include the Quadratic Integrate-Fire model [35] and, more recently, the Exponential Integrate-and-Fire (EIF) model [36] which has been shown to be in good agreement with experimental data [39]. The EIF model takes the form

$$\begin{array}{c} \tau \dot{v}=\mu -v+\delta_{T}e^{\left(v-v_{T}\right)/\delta_{T}}+\sigma \sqrt{2\tau }\xi \left(t\right) \end{array}$$ (49)

where the parameter *δ*_T_ sets the voltage range over which the spike initiates, *v*_T_ sets the spike-onset threshold and the other parameters have been previously defined earlier in the context of the LIF model. For the threshold the limit *v*_th_ → ∞ is typically taken but, practically, as long as *v*_th_ is significantly above *v*_T_ the behaviour is insensitive to its exact value. The reset has the same function as for the LIF model. Because of the non-linearity of the model many of the neuronal input-output functions are unavailable in analytical form; however, it is straightforward to solve the underlying differential equations numerically in the steady state [48] and for the first-passage-time density in the Fourier domain [49] (see Section 3.2 of that paper). For the latter calculation, replacing *iω* with *z* allows for all of the exponential expectations (Eq 1) required for the analytical results of this paper to be straightforwardly derived. Using this methodology, in Fig 5A–5E the results given in Fig 4 for the LIF are generalised to the EIF model, with the additional complexity of multiple contacts per presynaptic neuron included.

### From voltage moments to firing rate of IF neurons

It has been shown [44] that the postsynaptic firing rate of EIF neurons is surprisingly insensitive to (positive) temporal correlations when driven by coloured Gaussian noise for a given voltage mean and variance. This allows for a *matched-variance* approximation to be used in which the firing rate of a coloured-noise driven EIF neuron is approximated by its white noise equivalent but using the same subthreshold mean and variance. Eqs (32) and (42) can be used to find the voltage mean and variance and the EIF rate for white-noise drive is given in [36]. To test whether this approximation has validity, we consider the firing rate of an EIF neuron driven by depressing, stochastic synapses from a presynaptic population of EIF neurons. Following the same approach used in Fig 4 for LIF neurons, we covaried *v*_re_ and *σ* to get a range of spiking statistics in the presynaptic EIF population (see Eq 49 for the EIF model definition). In Fig 5A the firing rate of an EIF neuron as a function of the constant drive *μ* is shown for three different combinations of *v*_T_ − *v*_re_ and *σ*, with example time courses given in Fig 5B each having a rate 10Hz. In Fig 5C–5F the occupancy, voltage statistics and post-synaptic rate are plotted, as *v*_T_ − *v*_re_ and *σ* are simultaneosly linearly varied from their values on the blue and red curves in Fig 5A (with *μ* adapted so that the presynaptic rate is always 10Hz). A range of connectivity is considered by four combinations of *N* and *n* (as marked). As can be seen, the simulations agree well with the post-synaptic firing rate over a range of presynaptic firing patterns and forward connectivity choices. Hence, the matched variance approximation provides a fair account of the firing rate even in cases where the incoming fluctuations are negatively correlated, extending previous results [44].

### Generalisation to filtered synapses

It is worth noting it is fairly straightforward to generalise the voltage-variance calculations Eqs (29 and 42) to neurons receiving more biophysically shaped excitatory post-synaptic potentials (EPSPs). For example, if an isolated release event generates an EPSP of the form E(t) then, under the assumption of additivity,

$$\begin{array}{c} v=\left\langle v\right\rangle +\int_{-\infty }^{t}dt^{\prime }E\left(t-t^{\prime }\right)\sum_{i}(\chi_{i}\left(t^{\prime }\right)-\left\langle \chi \right\rangle ) \end{array}$$ (50)

becomes the generalisation of Eq (25) where the voltage mean is

$$\begin{array}{c} \left\langle v\right\rangle =\mu +N\left\langle \chi \right\rangle \;\int_{0}^{\infty }dt\;E\left(t\right). \end{array}$$ (51)

Following the same approach that led to Eq (29), the voltage variance in this case can be written

$$\begin{array}{c} \text{Var}\left(v\right)=N\left\langle \chi \right\rangle \;(\int_{0}^{\infty }dt\;E{\left(t\right)}^{2})\;(1+2p\int_{0}^{\infty }dz(G\left(z\right)-r{\left\langle x\right\rangle }_{\infty })\frac{\int_{0}^{\infty }dt\;E\left(t\right)E\left(t+z\right)}{\int_{0}^{\infty }dt\;E{\left(t\right)}^{2}})\;. \end{array}$$ (52)

If the form of the EPSP is modelled as a sum of multiple exponentials then the variance can again be expressed by Laplace transforms of *G*(*t*). For example, a two-exponential model for an EPSP

$$\begin{array}{c} E\left(t\right)=a\frac{\tau_{1}+\tau_{2}}{\tau_{1}-\tau_{2}}(e^{-t/\tau_{1}}-e^{-t/\tau_{2}}) \end{array}$$ (53)

has the integrals

$$\begin{array}{ccc} \;\int_{0}^{\infty }dt\;E{\left(t\right)}^{2}=\frac{a^{2}}{2}\left(\tau_{1}+\tau_{2}\right) & \;\text{and}\; & \frac{\int_{0}^{\infty }dt\;E\left(t\right)E\left(t+z\right)}{\int_{0}^{\infty }dt\;E{\left(t\right)}^{2}}=\frac{\tau_{1}e^{-z/\tau_{1}}-\tau_{2}e^{-z/\tau_{2}}}{\tau_{1}-\tau_{2}}. \end{array}$$ (54)

On substitution into the equation for the variance, and after identifying any Laplace transforms of *G*(*t*), this gives

$$\begin{array}{c} \text{Var}\left(v\right)=\frac{\left(\tau_{1}\;+\;\tau_{2}\right)Na^{2}}{2}\left\langle \chi \right\rangle \;(1+2p(\frac{\tau_{1}L_{G}\left(1/\tau_{1}\right)}{\tau_{1}-\tau_{2}}+\frac{\tau_{2}L_{G}\left(1/\tau_{2}\right)}{\tau_{2}-\tau_{1}}-r{\left\langle x\right\rangle }_{\infty }\left(\tau_{1}\;+\;\tau_{2}\right))\;)\;. \end{array}$$ (55)

The forms $L_{G}\left(z\right)$ are provided in the second equation of the pair (20) in terms of the Laplace transform of the first-passage-time density.

## Discussion

We have presented a series of novel analytical results that extend the level of biophysical detail incorporated in models of synaptic transmission. Non-Poissonian activity is commonly seen in vivo and can have a substantial effect on neuronal activity; relating this to synaptic dynamics allows for a more comprehensive understanding of the typical behaviour of plastic synapses. We have derived the exact spike-triggered mean vesicle occupancy, noting that it takes a particularly compact form when the input spike train is a renewal process, and highlighted the relationship between the spike-triggered mean and the overall level of vesicle occupancy, confirming the numerical results of Matveev and Wang (2000, [25]), de la Rocha and Parga (2005, [8]) and Reich and Rosenbaum (2013, [33]). We have derived the autocorrelations in vesicle release in terms of integral transforms, confirming the numerical results of Goldman et al (2002, [26]) and extending the analytical results of Goldman (2004, [29]) to account for the biophysically important case of probabalistic vesicle release.

The exact subthreshold voltage variance calculated from the neurotransmitter release autocorrelations is a potentially useful result and incorporates many biophysical details of autocorrelated input spikes, quantal effects, stochastic and cross-correlated vesicle release, and short-term plasticity. The relative effects of these biophysically relevant phenomena can now be quantitatively analysed [27, 50], providing insights into how changes in release-site number *n* arising from long-term plasticity [51] or in release probability *p* due to developmental changes [52–54] reshape the postsynaptic response to correlated spike trains.

The results for synaptic transmission when the presynaptic integrate-and-fire neuron is driven by short-time correlated noise processes are another important application, expanding the utility of the model to allow for study of synaptic dynamics alongside other known results concerning the firing rate [41, 42] and correlation structure [18, 43] of such neurons.

### Context

Many previous studies have examined how plastic, probabilistic and quantal synapses affect the statistics of patterned transmission through the synapse, and we now provide a selective overview. Vere-Jones (1966) [5] examined a model of a quantal, probabilistic synapse, finding that the release of neurotransmitter is more Poissonian than the afferent activity. Maass and Zador (1999) [27] considered the binary output of a single vesicle release site, investigating how triplets of incoming spikes corresponded to different release patterns under varying synaptic parameters. In particular they showed that dynamic synapses transmit bursts of spikes more reliably than static synapses and have enhanced computational power. Matveev and Wang (2000, [25]) numerically studied the effects of naturalistic presynaptic firing patterns on vesicle release, both with bursts generated by a two-state Markov model and long-time correlated trains. They found that spikes within a burst are suppressed by synaptic depression compared to isolated spikes, and, like Vere-Jones, that neurotransmitter release is more Poissonian than the incoming spikes. Goldman et al (2002, [26]) examined the transmission of doubly-stochastic Poissonian spike trains, constructed to reflect experimentally recorded neuronal bursting, finding again that dynamic synapses decorrelate afferent spike trains and so reduce coding redundancy across a broad range of synaptic parameters. As part of this study, the autocovariance of neurotransmitter release in response to temporally structured spike trains was calculated numerically. Goldman (2004, [29]) derived the information transmission efficiency of a depressing synapse analytically, finding the autocovariance of neurotransmitter release under the assumption of a synapse that reliably releases neurotransmitter whenever a vesicle is present. Using a model comprising a single neurotransmitter release site, de la Rocha et al (2002, [55]) showed that dynamic synapses were more effective transmitters of afferent signals only when the input is non-Poisson, analytically describing the distribution of synaptic release events when the input is a renewal process. They later [8] numerically studied the impact of temporal correlations on synapses containing multiple release sites, showing that bursty stimuli elicited fewer releases of neurotransmitter but that there could be a non-monotonic relationship between presynaptic and postsynaptic firing rates in the presence of input correlations and synaptic dynamics. Fuhrmann et al (2002, [56]) developed the stochastic quantal model used in this paper, capturing the same processes as the continuous phenomenological Tsodyks-Markram model of short-term plasticity [30], but focussed the initial analysis on Poisson spike trains. Ly and Tranchina (2009, [57]) considered numerically the transmission of temporally correlated spike trains across stochastic, but not dynamic, synapses and plotted the autocovariances in vesicle release, as well as the postsynaptic firing rate for renewal process inputs. Rosenbaum et al (2012, [12]) studied information transmission for Poissonian inputs, finding that the incorporation of stochastic quantal effects differentially affected information transmitted at different presynaptic rates. This paper approximated the auto- and cross covariances in neurotransmitter release in response to Poisson drive and these results were shown to be exact by Bird and Richardson (2014, [9]). Reich and Rosenbaum (2013, [33]) studied models of presynaptic spiking both more and less regular than a Poisson process, showing numerically that more regular firing patterns can increase the rate of vesicle release, thereby enhancing the fidelity and efficiency of signal transmission, whilst more irregular spike trains can lead to a decrease in neurotransmitter release. Zhang and Peskin (2015, [50]) developed the results of [12] on information transfer with unreliable dynamic synapses using a slightly simpler model of vesicle recovery, analytically studying the effects of a more general model of presynaptic spiking on neurotransmitter release rates and numerically simulating the effects on the postsynaptic membrane.

### Extensions

The matched-variance firing rate approximation in Fig 5 does not constitute a complete framework for treating recurrent networks of neurons with stochastic depressing synapses, because only the rate and not the full ISI statistics of the post-synaptic neuron were derived. However, it does suggest that some approximation scheme that goes beyond the first-order statistics of the ISI distribution might be used to analyse recurrent networks. This is currently a problem of great interest [21, 43, 58] given the strong effects correlated spike trains are acknowledged to have even across static synapses [18]. To include the components of vesicle-release autocorrelation arising from short-term depression, as modelled here, would increase physiological relevance and bring studies of output firing patterns into line with much of the literature on neuronal networks [31, 32, 59].

Another interesting extension is to account for the effect of spike-frequency adaptation currents on presynaptic firing. Adaptation is present across the nervous system [60] and can modulate responses to persistent activity by high-pass filtering and response selectivity [61–63]. These functional roles overlap with those attributed to synaptic depression [64], and there have been a number of recent studies on the interactions of short-term synaptic plasticity with slow adaptation mechanisms [65, 66]. A key feature of adaptation currents is the creation of correlations between interspike intervals [67], generating non-renewal spike trains. These correlations have recently been shown to take the form of a geometric series [68]. Eq (8) presents a way of deriving approximate results for the synaptic transmission for weakly correlated ISIs and suggests a promising avenue of research to go beyond renewal processes and study the effect of these two key short-term adaptive processes in neural circuits.

## Supporting information

S1 Code The Jupyter Notebook S1 Code generates and plots simulations of afferent spike trains, vesicle occupancy, and release for a single release site receiving a spike train with gamma-distributed ISIs for different values of *α*. It plots the ISI densities (Eq 3) and cumulative densities for different values of *α*. It plots the two occupancy means 〈*x*〉_∞_ and 〈*x*〉 (Eq 9) as a function of *α*, as well as their variances and the vesicle release mean 〈*χ*〉.
(JSON)

S2 Code The Jupyter Notebook S2 Code plots the temporal and spectral statistics of afferent spike trains and vesicle release for spike trains with gamma-distributed ISIs. It plots event-triggered rates, auto-covariances, and power spectra for the afferent spike train and vesicle release (Eqs 18 to 21). It plots the post-synaptic voltage mean (Eq 24), standard deviation (Eq 29), and coefficient of variation as a function of *α*.
(JSON)

S3 Code The Jupyter Notebook S3 Code generates and plots simulations of afferent spike trains, vesicle occupancy, and release for a single neuron with three independent release sites receiving a spike train with gamma-distributed ISIs for different values of *α*. It plots the correlation (Eq 34) and covariance (Eq 35) in occupancy as a function of *α* and the cross-covariance in vesicle release (Eq 39). It plots the post-synaptic voltage standard deviation (Eq 42) and coefficient of variation as a function of *α* for different numbers *n* of release sites per neuron.
(JSON)

S4 Code The Jupyter Notebook S4 Code plots the presynaptic firing rate of an LIF neuron receiving white noise drive for different spike threshold and reset values (Eq 45) [41]. It generates and plots presynaptic voltage traces and spike times for different parameter sets (Eq 44). It plots the spike-triggered occupancy 〈*x*〉_∞_ (Eq 48) and the post-synaptic voltage mean, variance, and coefficient of variation as a function of the threshold-reset difference for different presynaptic spike rates.
(JSON)

S5 Code The Jupyter Notebook S5 Code plots the presynaptic firing rate of an EIF neuron [36] receiving white noise drive for different spike threshold and reset values [49]. It generates and plots presynaptic voltage traces and spike times for different parameter sets (Eq 49). It plots the spike-triggered occupancy 〈*x*〉_∞_ and the post-synaptic voltage mean, variance, and simulated and estimated firing rates as a function of the threshold-reset difference for different presynaptic spike rates [44].
(JSON)
